# Draft genome sequence data on *Methanosarcina mazei* OFF1024 isolated from paddy field of Pondicherry, India

**DOI:** 10.1016/j.dib.2026.112715

**Published:** 2026-03-21

**Authors:** Munisamy Prathaban, Murugesan Sobanaa, Ragothaman Prathiviraj, S. Hari Krishna Kumar, George Seghal Kiran, Joseph Selvin, Laurent Dufossé

**Affiliations:** a Carbon Capture and Utilization Laboratory, Department of Microbiology, Pondicherry University, Pondicherry 605014, India; b Division of Bioinformatics and Systems Biology, Department of Biosciences, Rajagiri College of Social Sciences (Autonomous), Rajagiri P.O., Kalamassery, Kochi 683104, India; c Microbial Genomics Laboratory, Department of Microbiology, Pondicherry University, Pondicherry 605014, India; d Department of Food Science and Nutrition, Pondicherry University, Pondicherry 605014, India; e CHEMBIOPRO Lab (Chemistry & Biotechnology of Natural Products), University of La Réunion, ESIROI Agroalimentaire, 97744 Saint-Denis Cedex 9, Saint-Denis, Réunion, France

**Keywords:** Comparative genomics, Diazotrophic archaea, Draft genome, Methanogenesis, Methanosarcina mazei, Nitrogen fixation, Paddy field sediments

## Abstract

This article presents the draft genome sequence of *Methanosarcina mazei* strain OFF1024, isolated from paddy field sediments of Pondicherry, India. The genome was sequenced using the Illumina HiSeq PE150 platform and assembled *de novo* with MEGAHIT, resulting in a 4.07 Mb genome comprising 260 contigs with a GC content of 41.56%. Genome annotation identified 3459 coding sequences, 44 tRNA genes, and 3 rRNA genes. Comparative genomic analysis using TYGS confirmed the strain’s placement within the *M. mazei* species cluster, supported by high dDDH similarity to *M. mazei* C16 and *M. mazei* S-6. Functional gene mining revealed the nitrogen fixing gene set, including nitrogenase structural subunits, regulatory components, hydrogenases, and Fe–Mo cofactor biosynthesis genes, indicating the diazotrophic potential of this methanogen. The genome additionally encodes diverse methanogenesis pathways and stress-response mechanisms relevant to flooded agroecosystems. This dataset provides a valuable genomic resource for studying methanogenic archaea in paddy soils, supporting future research on methane cycling, archaeal ecology, nitrogen fixation, and bioenergy applications.

Specification Table

**Table:** 

Subject	Applied Microbiology and Biotechnology
Specific subject area	Genomics
Type of data	Whole genome sequence of *Methanosarcina mazei* strain OFF1024 raw and analyzed, represented as tables and figures
Data collection	Genomic DNA was isolated using a DNA extraction kit from HiMedia, India. The whole genome was sequenced at Macrogen Inc. (Seoul, South Korea) using the Illumina HiSeq PE150 platform. The quality check was done in FastQC software version 0.11.9, to check the adapter sequence and low-quality region in the sequence, Fastp tool was used to remove the adapter region. After pre-processing, the sequence was assembled using MEGAHIT assembler and the genome statistics were assessed by the Quast tool. The assembled genome circular plot was constructed using the Proksee server and the assembled genome was annotated and gene predictions were carried out using Prokka version 1.14.6.
Data source location	M. mazei strain OFF1024 was isolated from paddy field sediment samples taken from the Paddy cultivation field of Pondicherry in India (11.900476° N, 79.812477° E).
Data accessibility	Whole Genome data was deposited in the National Center for Biotechnology Information (NCBI) GenBank database with the following accession number NZ_JBLVWA000000000.1. The deposited draft genome data available at https://www.ncbi.nlm.nih.gov/nuccore/NZ_JBLVWA000000000.1
Related research article	None

## 1 Value of the Data

• The draft genome of *Methanosarcina mazei* OFF1024 expands the genomic representation of *M. mazei* strains isolated from tropical paddy field ecosystems in Pondicherry, India, a region currently underrepresented in publicly available genome datasets.
• The genome encodes complete methyl-coenzyme M reductase complex and associated methanogenesis pathways alongside nitrogen fixation-related genes, providing a genomic framework for investigating carbon–nitrogen coupling in diazotrophic methanogens under nitrogen-limited conditions.
• The availability of assembly metrics, quality assessment (97.21% completeness; 0.54% contamination), and KEGG-based functional annotation enhances reproducibility and facilitates future comparative and meta-genomic studies within the genus *Methanosarcina*.
• The genomic information can support cross-boundary microbiome research, particularly in linking archaeal community functions with soil physicochemical parameters influencing greenhouse gas fluxes in tropical rice ecosystems.

## 2 Background

Methanogenic archaea play a crucial role in the terminal stages of anaerobic organic matter degradation, producing methane as a metabolic end product [[1], [2], [3], [4]]. Among them, *M. mazei* represents one of the most metabolically versatile methanogens, capable of utilizing multiple substrates, including acetate, methanol, methylamines, and hydrogen–carbon dioxide, for methanogenesis [5]. This versatility allows *M. mazei* to thrive in diverse anaerobic environments, including wetlands, sediments, and paddy soils [6]. Rice paddy ecosystems are recognized as major anthropogenic sources of atmospheric methane emissions due to their waterlogged and anoxic conditions that favor methanogenic activity [1]. The abundance and community structure of methanogens in such systems are highly influenced by soil characteristics, redox potential, and the availability of organic substrates [7]. Understanding the genomic features of dominant methanogens such as *M. mazei* is essential for elucidating their ecological roles and adaptive mechanisms under fluctuating field conditions.

In addition to methanogenesis, certain members of the methanogens possess genes encoding nitrogenase complexes that enable biological nitrogen fixation under nitrogen-limited conditions [8]. Nitrogen fixation in methanogenic archaea is energetically demanding and tightly regulated, requiring coordination between nitrogenase structural genes (nifHDK), Fe–Mo cofactor biosynthesis pathways, and hydrogenase-mediated electron transfer systems [9]. In flooded paddy soils, where redox conditions fluctuate and inorganic nitrogen availability can be limited, diazotrophic methanogens may contribute to both nitrogen turnover and methane production. The coexistence of methanogenesis and nitrogen fixation pathways in the same organism represents an ecologically significant metabolic integration that influences carbon–nitrogen coupling in anaerobic agroecosystems [10,11]. Therefore, genome-level characterization of nitrogen fixation potential in *M. mazei* strains from paddy fields provides important insight into their adaptive strategies and possible contributions to nutrient cycling.

In this framework, strain OFF1024 of *M. mazei* was isolated from paddy field sediments of Pondicherry, India, a region categorized by tropical climatic conditions and cyclic wet-to-dry cultivation regimes. The draft genome of *M. mazei* OFF1024 provides valuable insights into genes associated with methane metabolism, environmental stress tolerance, nitrogen fixation and cross-domain microbial interactions within paddy agroecosystems. This genomic information will serve as a foundational reference for comparative analyses of methanogenic archaea and may support the development of strategies aimed at mitigating methane emissions and improving nitrogen fixation in rice cultivation.

## 3 Data Description

**Fig. 1:**
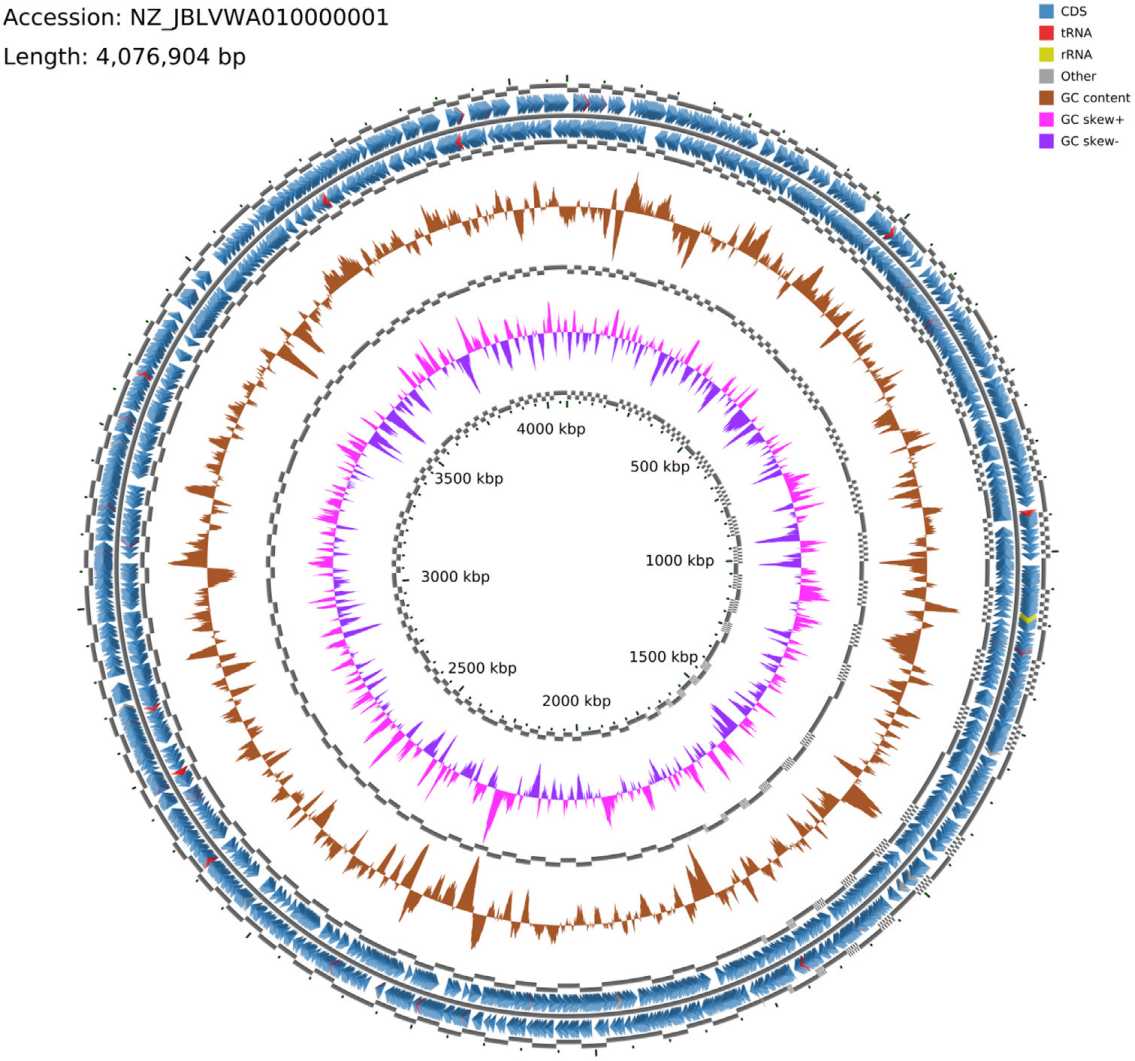
Circular genome map of *M. mazei* OFF1024 (Accession: NZ_JBLVWA0100000001; genome size: 4076,904 bp). The outer rings display coding DNA sequences annotated on the forward and reverse strands, followed by annotated tRNA and rRNA genes along with other genomic features. The inner rings show GC content variation across the genome, and the innermost rings depict GC skew, where positive and negative values indicate fluctuations in nucleotide composition that may correspond to replication origin and terminus regions. The concentric scale marks represent genome position in kilobase pairs (kbp). This circular representation highlights gene distribution, structural organization, and compositional characteristics across the complete genome of *M. mazei* OFF1024.

The dataset reports the draft genome sequence of *M. mazei* OFF1024, an archaeal isolate of biotechnological importance due to its methanogenesis and nitrogen fixation capabilities. Sequencing generated a total of 2.46 million reads (1.23 million paired-end reads; 2 × 150 bp), yielding 369,475,600 bases of raw data with a coverage depth of 90×. *De novo* assembly was performed using MEGAHIT with default k-mer parameters and a minimum contig length threshold of 500 bp. *De novo* assembly yielded a genome size of 4076,904 bp distributed across 260 contigs with an N50 of 26,878 bp and an L50 of 51. Genome quality assessment was performed using CheckM (v1.2.4) with the *Methanosarcina* lineage-specific marker set. The analysis estimated a genome completeness of 97.21% and contamination of 0.54%, indicating a high-quality draft genome suitable for comparative genomic and functional analyses. The average GC content of the genome was 41.56%. Genome annotation predicted 3459 protein-coding sequences (CDS), 44 tRNA genes, and 3 rRNA genes, along with 2205 hypothetical proteins (Table 1). A circular genome map of *M. mazei* OFF1024 illustrating the global genomic architecture, gene orientation, RNA features, GC content, and GC skew distribution is shown in Fig. 1. Genome-based comparative analysis revealed that *M. mazei* OFF1024 shows the highest genomic relatedness to members of the *M. mazei* species group. Strain OFF1024 exhibited the highest dDDH similarity to *Methanosarcina mazei* C16 (dDDH_d4 = 90.5%) and *M. mazei* S-6 (dDDH_d4 = 85.5%), well above the 70% species delineation threshold [[12], [13], [14]]. Comparisons with other *Methanosarcina* members demonstrated variable genomic relatedness. Although strain OFF1024 exhibited a dDDH value of 81.4% with *M. soligelidi* SMA-21, TYGS type-based species clustering analysis places *M. soligelidi* within a distinct species cluster separate from *M. mazei*. More distantly related species, including *M. horonobensis, M. subterranea, M. hadeiensis*, and *M. acetivorans*, showed substantially lower dDDH values (26–29%), well below the species delineation threshold. High dDDH values (>85%) and minimal *G* + *C* content differences (<0.2%) between OFF1024 and reference *M. mazei* strains confirm that OFF1024 is assigned to the species *Methanosarcina mazei*. These reduced similarities fall well below species-level thresholds, further confirming the taxonomic assignment of strain OFF1024.

**Table 1:** Genome statistics of *M. mazei* OFF1024.

Features	Count
Contigs	260
GC content	41.56
Genome length (bp)	40,76,904
Contig L50	51
Contig N50	26,878
CDS	3459
tRNA	44
rRNA	3
Hypothetical proteins	2205

Functional analysis revealed the presence of a comprehensive gene repertoire associated with biological nitrogen fixation (Table 2). Core nitrogenase components, including genes encoding the iron protein and the molybdenum-iron catalytic subunits responsible for the reduction of atmospheric N₂ to NH₃, were detected. The genome also encodes regulatory proteins involved in nitrogen metabolism control, proteins supporting Fe–Mo cofactor biosynthesis required for nitrogenase activity, and periplasmic [NiFeSe] hydrogenases that likely contribute to electron transfer during energy-intensive diazotrophic processes. Additional features include transcriptional regulators linked to metal homeostasis required for nitrogenase function, and tRNA-associated elements involved in nitrogen assimilation pathways [15].

**Table 2:** Distribution of genes encoding nitrogen fixation identified in the genome of *M. mazei* OFF1024.

Biology	Function relevance	No. of genes
Nitrogenase iron protein	Core nitrogen-fixing gene (essential for N₂ → NH₃ conversion)	3
Nitrogenase molybdenum-iron protein	Catalytic core of nitrogenase complex that reduces atmospheric N₂ to NH₃ using the Fe–Mo cofactor during biological nitrogen fixation	4
Nitrogen regulatory protein	Regulates nitrogen metabolism by controlling transcription of nitrogen assimilation and nitrogen fixation genes	4
Periplasmic [NiFeSe] hydrogenase	Supports electron transfer, often linked to diazotrophic energy metabolism	4
FeMo cofactor biosynthesis protein	Involved in the assembly of the Fe–Mo cofactor, an essential metallocluster required for nitrogenase catalytic activity	1
tRNA-Gln	Not a nif gene but related to nitrogen assimilation	2
Transcriptional regulator	Indirectly linked to metal regulation important for nitrogenase cofactors (Fe/Mo)	3

KEGG-based functional annotation (eggNOG mapper) identified a complete set of methanogenesis-related genes in *M. mazei* OFF1024. Core components of the methyl-coenzyme M reductase complex (mcrA, mcrB, mcrG), which catalyze the terminal step of methane formation, were detected (Table 3). Genes encoding formylmethanofuran dehydrogenase subunits, supporting the hydrogenotrophic CO₂ reduction pathway, were also present. Additionally, methylotrophic pathway genes including trimethylamine methyltransferase and associated methyl group carrier proteins were identified (Table 3). These results confirm the genomic capacity of strain OFF1024 to utilize multiple methanogenic substrates consistent with the metabolic versatility of the *M. mazei* species.

**Table 3:** Distribution of methanogenesis-encoding genes identified in *M. mazei* OFF1024.

Pathway category	Enzyme / Functional annotation	Gene copies detected	Functional role
Terminal Methane Formation	Methyl-coenzyme M reductase alpha subunit (*McrA*)	1	Catalyzes final step of methane formation
	Methyl-coenzyme M reductase beta subunit (*McrB*)	1	Part of MCR catalytic complex
	Methyl-coenzyme M reductase gamma subunit (*McrG*)	1	Part of MCR catalytic complex
Hydrogenotrophic CO₂ reduction pathway	Formylmethanofuran dehydrogenase subunit A	2	Initial step of CO₂ reduction
	Formylmethanofuran dehydrogenase subunit B	3	CO₂ reduction complex component
	Formylmethanofuran dehydrogenase subunit D/domain	3	CO₂ reduction complex component
Methylotrophic pathway (Methylamine utilization)	Trimethylamine methyltransferase (MTTB-type)	4	Methylamine-dependent methanogenesis
	Methyl group carrier protein	3	Transfers methyl group to coenzyme M

**Fig. 2:**
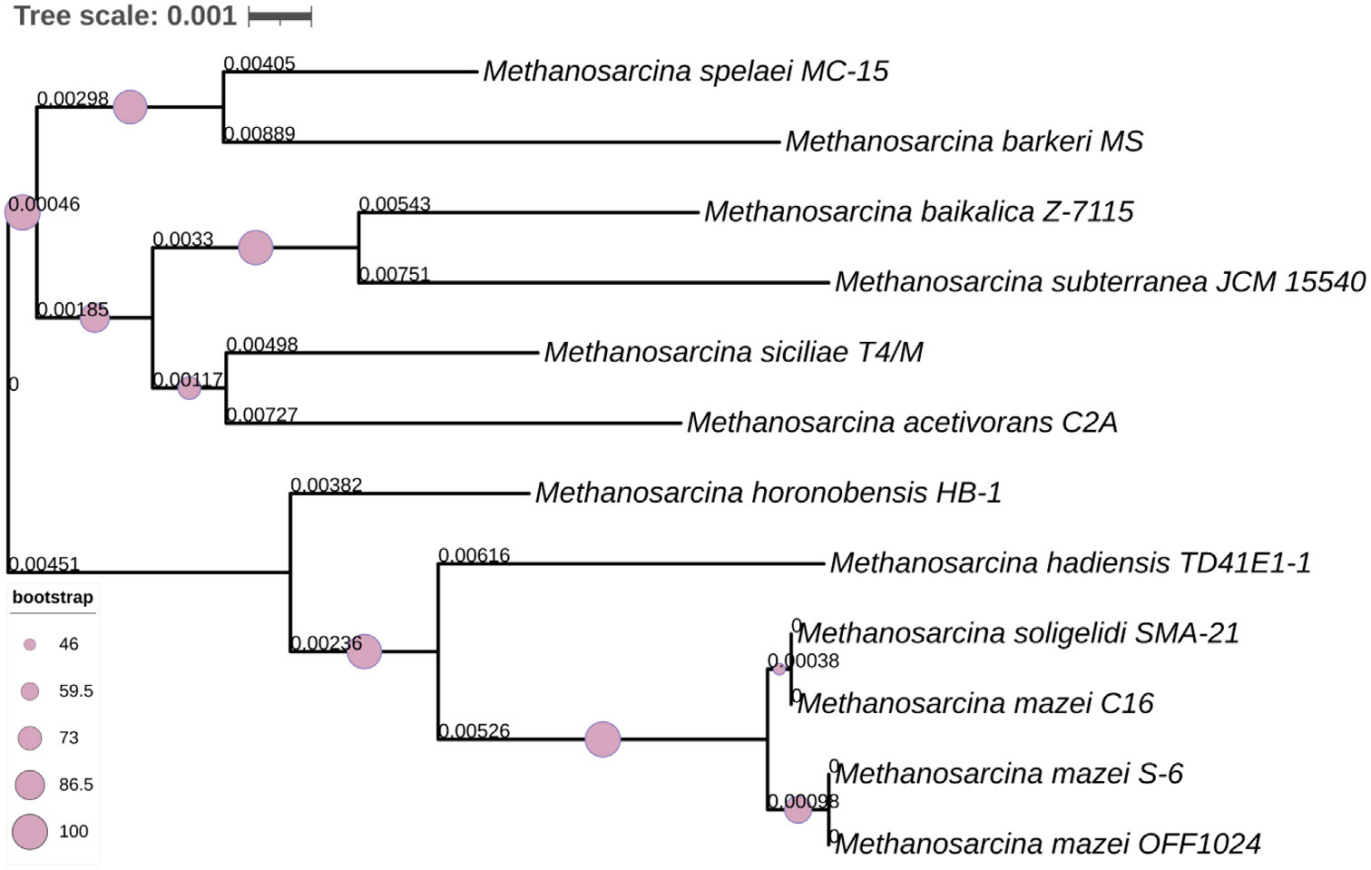
16S rRNA gene-based phylogenetic tree relationship among *Methanosarcina* species. The phylogenetic tree was constructed using the Neighbor-Joining algorithm with 10,000 bootstrap replicates. Branch lengths correspond to genetic distances, with the tree scale (0.001) indicating the number of nucleotide substitutions per site.

**Fig. 3:**
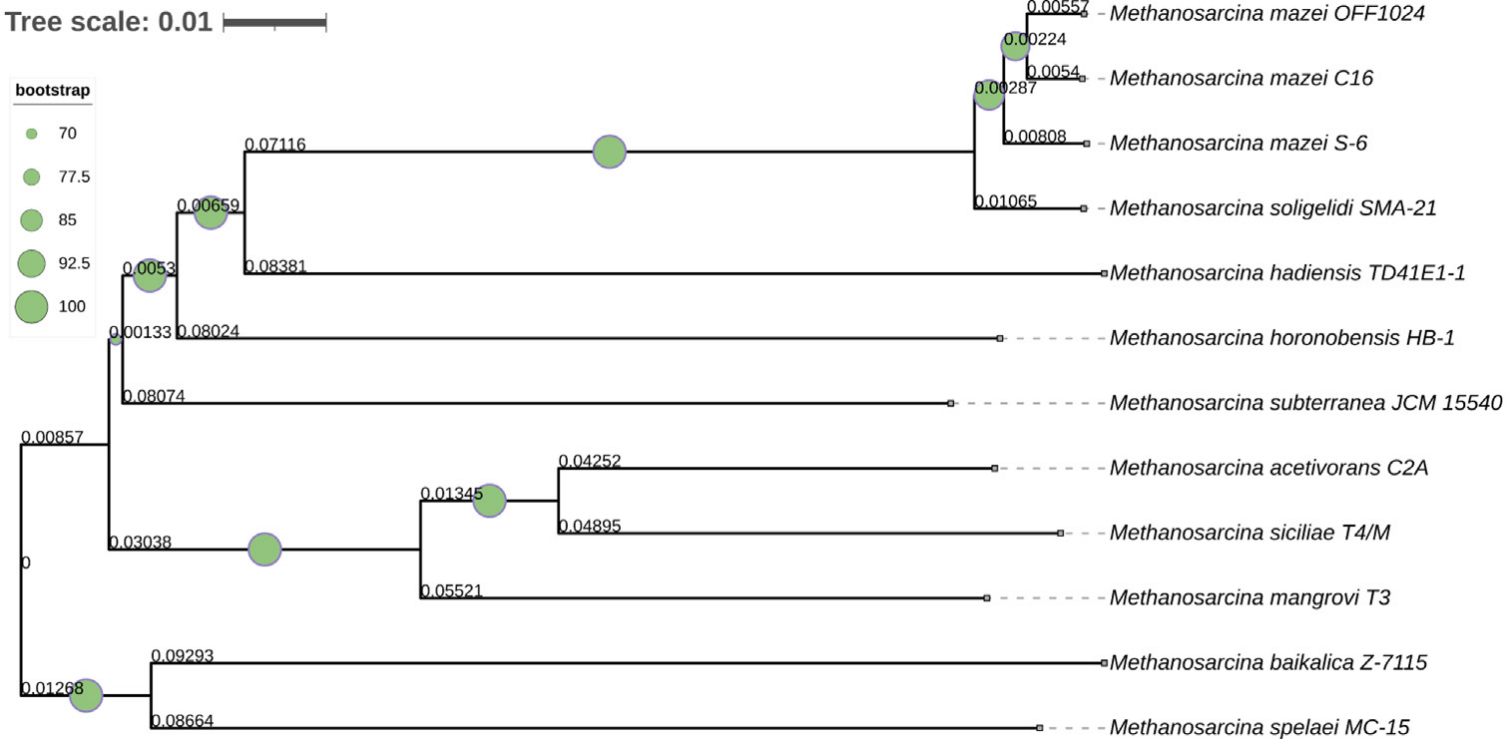
Genome-based phylogenomic tree of *M. mazei* OFF1024. The tree illustrates the phylogenomic relationship of *M. mazei* OFF1024 with representative genomes of *Methanosarcina* species and subspecies. The phylogeny was constructed using whole-genome sequence data, and branch support values (bootstrap percentages) are indicated at the nodes.

The 16S rRNA gene-based phylogeny (Fig. 2) positions *M. mazei* OFF1024 in close proximity to *M. mazei* S-6, supported by strong bootstrap values, indicating conserved evolutionary relationships at the ribosomal gene level. Whole-genome phylogenomic analysis (Fig. 3) further resolved strain-level relationships using genome-wide sequence comparisons. In the genome-based tree, OFF1024 clustered tightly with reference *M. mazei* genomes, particularly strains C16 and S-6, forming a well-supported clade (bootstrap support > 60%). This topology is consistent with the digital DNA–DNA hybridization (dDDH) values, which exceed the 70% species threshold, confirming species-level assignment. While Fig. 3 illustrates phylogenomic relationships based on genome-wide distances, the corresponding quantitative dDDH values supporting species delineation are summarized in the TYGS output. Together, these analyses demonstrate both phylogenetic coherence and strain-level divergence within the *M. mazei* clade. Compared with reference *M. mazei* strains S-6 (4.14 Mb, 3347 proteins) and C16 (4.16 Mb, 3371 proteins), strain OFF1024 exhibits a slightly reduced genome size (4.07 Mb) but a comparable coding potential (3459 CDS). Nitrogenase structural genes were conserved across *M. mazei* representatives, indicating preservation of diazotrophic capability within the species. The assembled genome has been deposited in GenBank under the accession NZ_JBLVWA000000000.1, enabling public access for further comparative genomics, evolutionary analysis, and functional investigations of methanogenesis-linked nitrogen-fixing archaeal systems.

The draft genome of *M. mazei* OFF1024 reveals key genes associated with methanogenesis and nitrogen metabolism that are consistent with the known genomic and physiological traits of the *Methanosarcina* genus. Previous studies have documented the metabolic versatility of *M. mazei*, including its ability to utilize multiple substrates for methanogenesis and its association with nitrogen fixation pathways in anoxic environments [16]. The detection of nitrogenase structural genes and accessory proteins in OFF1024 agrees with earlier reports showing that several *Methanosarcina* species harbor *nif*-like genes and can participate in nitrogen acquisition under nutrient-limited conditions [15]. Regulatory proteins, Fe–Mo cofactor biosynthesis components, and hydrogenase systems identified in this genome also correspond to previously documented nitrogen metabolism mechanisms in nitrogen-fixing methanogens [6]. Overall, the dataset contributes genomic insight into archaeal nitrogen fixation, hydrogen metabolism, and methanogenic regulatory networks. This study contributes valuable genomic insight into nitrogen fixation in methanogenic archaea, supporting future research in carbon–nitrogen cycling, microbial ecology, and bioengineering applications involving sustainable methane production and diazotrophic metabolism.

## 4 Experimental Designs, Materials and Methods

Sediment samples were collected from a paddy field at 11.940625 N, 79.744643 E, using sterile corers at a depth of 10–20 cm below the surface. Samples were transferred to sterile screw cap bottles pre-flushed with N₂ gas, sealed immediately to maintain anaerobic conditions, and transported at 4 °C for processing. For enrichment of methanogenic archaea, bicarbonate yeast tryptone (BCYT) medium was prepared, dispensed into serum vials, sealed with butyl rubber stoppers, and flushed with N_2_, sterilized at 121 °C for 20 mins. After sterilization media was cooled down and added with methanol (1% V/V), trace elements, vitamins solution and reducing agent and flushed with N_2_. Sediment (1 g) was inoculated into enrichment vials and incubated anaerobically at 37 °C for 10–14 days until turbidity was observed. Cultures were purified by serial dilution and the roll tube technique under N_2_ atmosphere [2].

### 4.1 Genomic DNA extraction from M. mazei OFF1024

*M. mazei* OFF1024 was cultured anaerobically in BCYT medium containing methanol (1%V/V) under N_2_ atmosphere at 37 °C until mid-log phase. Cells were collected by centrifugation at 8000 × *g* for 10 min, and genomic DNA was extracted using a HiMedia DNA extraction kit (HiMedia, India) according to the manufacturer’s protocol [17].

### 4.2 Whole genome sequencing and assembly

The genomic DNA of *M. mazei* OFF1024 was sequenced at Macrogen Inc. (Seoul, South Korea) using the Illumina HiSeq platform with paired-end 150 bp chemistry (PE150). The raw sequencing reads were subjected to initial quality assessment using FastQC v0.11.9 to evaluate base quality scores [18], GC distribution, adapter contamination, and sequence duplication levels. Adapter trimming and removal of low-quality reads were performed using Fastp, ensuring improved read fidelity for downstream assembly [19]. High-quality filtered reads were assembled *de novo* using the MEGAHIT assembler, a short-read optimized metagenomic and genomic assembly tool [20,21]. Assembly quality metrics, including contig statistics, N50, L50, GC content, and genome length, were evaluated using QUAST (Quality Assessment Tool for Genome Assemblies) [22]. Assembly quality and lineage-specific completeness were further evaluated using CheckM v1.2.4 with the Methanosarcina marker set, providing estimates of genome completeness and contamination [23]. The structural organization and genomic feature distribution were visualized through a circular genome map constructed using the Proksee web server. Genome annotation and gene prediction were performed using Prokka v1.14.6 and eggNOG mapper v2.1.13 enabling the identification of coding sequences, ribosomal and transfer RNA genes, functional elements, and assignment of preliminary gene annotations [24]. The resulting genome assembly and annotation outputs were used for downstream comparative genomics and functional analysis.

### 4.3 Phylogenetic tree construction

The assembled genome was submitted to the Type (Strain) Genome Server (TYGS; https://tygs.dsmz.de) for whole-genome–based taxonomic assessment using the most recent analytical framework [25]. Nomenclature and taxonomic references were obtained from the List of Prokaryotic names with Standing in Nomenclature (LPSN; https://lpsn.dsmz.de) [24]. Closest type strains were identified through two TYGS-integrated approaches: (i) MASH comparison of the query genome against all available type strain genomes to retrieve the ten nearest neighbors based on lowest MASH distances [26], and (ii) RNAmmer-based extraction of 16S rRNA sequences [27] followed by BLAST against 23,787 type strain entries, from which the top 50 hits were subjected to distance estimation using Genome BLAST Distance Phylogeny (GBDP) with the coverage algorithm and distance formula d5 [28]. The ten most closely related strains were selected for further comparison. Phylogenomic inference was performed in TYGS using GBDP with the trimming algorithm and distance formula d5, incorporating 100 distance replicates to ensure stable tree topology [29]. The d5 distance formula was used alongside the "trimming" algorithm [27] to calculate intergenomic distances. For each pairwise comparison, 100 replicated distance calculations were performed. Estimates for digital DNA-DNA hybridization (dDDH) and their associated confidence intervals were generated following the guidelines of the GGDC 4.0 platform [27,29].

## Limitations

This study presents a high-quality draft genome assembled using short-read Illumina sequencing at approximately 90× coverage. Although genome completeness was high (97.21%) with minimal contamination (0.54%), the assembly remains fragmented (260 contigs) due to the absence of long-read sequencing, which may limit resolution of repetitive regions or structural rearrangements. Functional predictions, including methanogenesis and nitrogen fixation potential, are currently based on in silico annotation. The organism is presently being investigated for its nitrogen fixation capacity and methane production under nitrogen-limited conditions, and experimental validation of these metabolic traits is ongoing. The raw sequencing reads are securely archived and will be made publicly available upon completion of the ongoing functional study to ensure full data accessibility and reproducibility.

## Ethics Statement

This study did not involve any experiments on human participants or animals. All data were obtained through computational analyses. Hence, ethical approval was not required for this work.

## CRediT Author Statement

**Prathaban Munisamy:** Validation, Conceptualization, Methodology**,** Writing – original draft, Writing – review & editing**; Sobanaa Murugesan:** Writing – original draft, Methodology, Writing – review & editing**; Ragothaman Prathiviraj:** Visualization, Writing – review & editing, Data curation; **S. Hari Krishna Kumar**: Visualization, Writing – review & editing, Data curation; **George Seghal Kiran**: Visualization, Writing – review & editing**; Joseph Selvin**^:^ Supervision, Administration, resource, Writing – review & editing **Laurent Dufossé:** Supervision, Conceptualization, Writing – original draft, Writing – review & editing.

## Declaration of Generative AI and AI-assisted Technologies in the Writing Process

During the preparation of this work, the authors used Chat GPT and Quilbot for paraphrasing and language improvement.

## Data Availability

NCBI)Methanosarcina isolated from paddy field applied with organic fertilizers (Original data).

## Appendix Supplementary materials

Image, application 1
