# Supplementary material for: Draft genome sequence data on Methanosarcina mazei OFF1024 isolated from paddy field of Pondicherry, India
Source: Data Brief. 2026 Mar 21;66:112715. doi: 10.1016/j.dib.2026.112715 (PMC13054258; doi:10.1016/j.dib.2026.112715)
Supplement: Supplementary file 1 [file mmc1.pdf]

TYPE (STRAIN) GENOME SERVER

JOB SUMMARY

PRINT DATE: 2025-11-14 10:57:11 +0100

JOB ID: 06f2c43d-6d38-41e0-9850-847f24c5a424

RESULT PAGE: https://tygs.dsmz.de/user_results/show?guid=06f2c43d-6d38-41e0-9850-847f24c5a424

Table 1: Phylogenies

Publication-ready versions of both the genome-scale GBDP tree and the 16S rRNA gene sequence tree can be customized and exported either in SVG (vector graphic) or PNG format from within the phylogeny viewers in your TYGS result page. For publications the **SVG format is recommended** because it is lossless, always keeps its high resolution and can also be easily converted to other popular formats such as PDF or EPS. Please follow the link provided above!

Table 2: Identification

The below list contains the result of the TYGS species identification routine.

Explanation of remarks that might occur in the below table:

remark [R1]: The TYGS type strain database is automatically updated on an almost daily basis. However, if a particular type strain genome is not available in the TYGS database, this can have several reasons which are detailed in the FAQ. You can request an extended 16S rRNA gene analysis via the 16S tree viewer found in your result page to detect **not yet genome-sequenced** type strains relevant for your study.

remark [R2]: > 70% dDDH value (formula d_4) and (almost) minimal dDDH values for gene-content formulae d_0 and d_6 indicate a potentially unreliable identification result and should thus be checked via the 16S rRNA gene sequence similarity. Such strong deviations can, in principle, be caused by sequence contamination.

remark [R3]: G+C content difference of > 1 % indicates a potentially unreliable identification result because within species G+C content varies no more than 1 %, if computed from genome sequences (PMID: 24505073).

Strain	Conclusion	Identification result	Remark
**'Methanosarcina mazei OFF0124'**	belongs to known species	*Methanosarcina mazei*	

Table 3: Pairwise comparisons of user genomes vs. type-strain genomes

The following table contains the pairwise dDDH values between your user genomes and the selected type-strain genomes. The dDDH values are provided along with their confidence intervals (C.I.) for the three different GBDP formulas:

- formula d_0 (a.k.a. GGDC formula 1): length of all HSPs divided by total genome length
- formula d_4 (a.k.a. GGDC formula 2): sum of all identities found in HSPs divided by overall HSP length
- formula d_6 (a.k.a. GGDC formula 3): sum of all identities found in HSPs divided by total genome length

Note: Formula d_4 is independent of genome length and is thus robust against the use of incomplete draft genomes. For other reasons for preferring formula d_4, see the FAQ.

Query	Subject	d_0	C.I. d_0	d_4	C.I. d_4	d_6	C.I. d_6	Diff. G+C Percent
**'Methanosarcina mazei OFF0124.fasta'**	*Methanosarcina frisia* C16	90.1	[86.9 - 92.6]	90.5	[88.3 - 92.4]	92.7	[90.4 - 94.5]	0.08
**'Methanosarcina mazei OFF0124.fasta'**	*Methanosarcina mazei* S-6	88.0	[84.5 - 90.8]	85.5	[82.8 - 87.8]	90.4	[87.7 - 92.5]	0.15
**'Methanosarcina mazei OFF0124.fasta'**	*Methanosarcina soligelidi* SMA-21	84.1	[80.3 - 87.3]	81.4	[78.5 - 84.0]	86.6	[83.5 - 89.2]	0.07
**'Methanosarcina mazei OFF0124.fasta'**	*Methanosarcina hadiensis* TD41E1-1	31.1	[27.7 - 34.7]	27.9	[25.5 - 30.4]	29.3	[26.3 - 32.4]	0.4
**'Methanosarcina mazei OFF0124.fasta'**	*Methanosarcina horonobensis* HB-1	27.5	[24.1 - 31.1]	27.4	[25.0 - 29.9]	26.3	[23.4 - 29.4]	0.26
**'Methanosarcina mazei OFF0124.fasta'**	*Methanosarcina subterranea* JCM 15540	24.7	[21.4 - 28.4]	27.3	[24.9 - 29.8]	24.0	[21.1 - 27.1]	0.39
**'Methanosarcina mazei OFF0124.fasta'**	*Methanosarcina acetivorans* C2A	20.8	[17.6 - 24.4]	26.1	[23.8 - 28.6]	20.5	[17.7 - 23.5]	1.11
**'Methanosarcina mazei OFF0124.fasta'**	*Methanosarcina siciliae* T4/M	20.9	[17.7 - 24.5]	25.6	[23.2 - 28.0]	20.5	[17.8 - 23.6]	1.37
**'Methanosarcina mazei OFF0124.fasta'**	*Methanosarcina spelaei* MC-15	15.4	[12.5 - 18.9]	24.2	[21.8 - 26.6]	15.6	[13.1 - 18.5]	2.8
**'Methanosarcina mazei OFF0124.fasta'**	*Methanosarcina barkeri* MS	15.5	[12.6 - 18.9]	23.6	[21.3 - 26.1]	15.6	[13.1 - 18.5]	2.38
**'Methanosarcina mazei OFF0124.fasta'**	*Methanosarcina baikalica* Z-7115	16.4	[13.4 - 19.9]	22.8	[20.6 - 25.3]	16.4	[13.9 - 19.4]	0.82

Table 4: Strains in your dataset

Joint dataset of automatically determined closest type strains (if this mode was chosen), manually selected type strains (if selected accordingly) and the provided user strains, if provided (marked in yellow).

Strain	Authority	Other deposits	Synonyms	Base pairs	Percent G+C	No. proteins	Goldstamp	Bioproject accession	Biosample accession	Assembly accession	IMG OID
Methanosarcina spelaei MC-15	Ganzert et al. 2014	DSM 26047; JCM 18469	*Methanosarcina spelaei*	4967 267	38.8	3818	Gp0322643	PRJNA300715	SAMN04229036	GCA_002287235	
Methanosarcina mazei S-6	(Barker 1936) Mah and Kuhn 1984	DSM 2053; VKM B-1636; not cultivated originally; OCM 26	*Methanococcus mazei*; *Methanosarcina mazei*	4142 816	41.4	3347	Gp0074654	PRJNA230947	SAMN03074583	GCA_000970205	
Methanosarcina siciliae T4/M	(Stetter and König 1989) Ni et al. 1994 emend. Elberson and Sowers 1997	ATCC BAA-931; DSM 3028; OCM 156	*Methanolobus siciliae*; *Methanosarcina siciliae*	5017 558	42.9	3971	Gp0074660	PRJNA230953	SAMN03074589	GCA_000970085	
Methanosarcina barkeri MS	Schnellen 1947 emend. Maestrojuán et al. 1992	ATCC 43569; DSM 800; JCM 10043; NBRC 100474; VKM B-1635; OCM 38	*Methanosarcina barkeri*	4574 907	39.2	3518	Gp0074646	PRJNA230939	SAMN03074575	GCA_000970025	
Methanosarcina horonobensis HB-1	Shimizu et al. 2011	DSM 21571; JCM 15518; NBRC 102577	*Methanosarcina horonobensis*	5018 607	41.3	4095	Gp0074649	PRJNA230942	SAMN03074578	GCA_000970285	
Methanosarcina soligelidi SMA-21	Wagner et al. 2013	DSM 26065; JCM 18468	*Methanosarcina soligelidi*	4064 496	41.5	3453	Gp0032041	PRJNA210206	SAMN02745719	GCA_000744315	
Methanosarcina frisia C16	(Blotevogel et al. 1986) Blotevogel and Fischer 1989	ATCC 43340; DSM 3318; OCM 98	*Methanococcus frisius*; *Methanosarcina frisia*	4166 241	41.5	3371	Gp0074652	PRJNA230945	SAMN03074581	GCA_000970245	

Strain	Authority	Other deposits	Synonyms	Base pairs	Percent G+C	No. proteins	Goldstamp	Bioproject accession	Biosample accession	Assembly accession	IMG OID
Methanosarcina baikalica Z-7115	Zhilina et al. 2025	JCM 39438; VKM B-3565	*Methanosarcina baikalica*	3734 836	40.8	3150		PRJNA224116	SAMN37284058	GCF_031461075	
Methanosarcina hadiensis TD41E1-1	Giménez et al. 2024	DSM 117368	*Methanosarcina hadiensis*	4338 376	41.2	3645		PRJNA224116	SAMN37575900	GCF_046529725	
Methanosarcina acetivorans C2A	Sowers et al. 1986	ATCC 35395; DSM 2834; JCM 12185; NBRC 100939; OCM 95	*Methanosarcina acetivorans*	5751 492	42.7	4540	Gp0000695	PRJNA290	SAMN03081414	GCA_000007345	638154508
Methanosarcina subterranea JCM 15540	Shimizu et al. 2015	DSM 22503; NBRC 102578; HC-2	*Methanosarcina subterranea*	5061 919	41.2	4300		PRJDB20344	SAMD00887336		
Methanosarcina mazei OFF0124.fasta				4080 276	41.6	3512					

The genome sequence data were uploaded to the Type (Strain) Genome Server (TYGS), a free bioinformatics platform available under https://tygs.dsmz.de, for a whole genome-based taxonomic analysis [1]. The analysis also made use of recently introduced methodological updates and features [2,3]. Information on nomenclature, synonymy and associated taxonomic literature was provided by TYGS's sister database, the List of Prokaryotic names with Standing in Nomenclature (LPSN, available at https://lpsn.dsmz.de) [2,3]. The results were provided by the TYGS on 2025-11-07. The TYGS analysis was subdivided into the following steps:

Determination of closely related type strains

Determination of closest type strain genomes was done in two complementary ways: First, all user genomes were compared against all type strain genomes available in the TYGS database via the MASH algorithm, a fast approximation of intergenomic relatedness [4], and, the ten type strains with the smallest MASH distances chosen per user genome. Second, an additional set of ten closely related type strains was determined via the 16S rDNA gene sequences. These were extracted from the user genomes using RNAmmer [5] and each sequence was subsequently BLASTed [6] against the 16S rDNA gene sequence of each of the currently 23833 type strains available in the TYGS database. This was used as a proxy to find the best 50 matching type strains (according to the bitscore) for each user genome and to subsequently calculate precise distances using the Genome BLAST Distance Phylogeny approach (GBDP) under the algorithm 'coverage' and distance formula d_5 [7]. These distances were finally used to determine the 10 closest type strain genomes for each of the user genomes.

Pairwise comparison of genome sequences

For the phylogenomic inference, all pairwise comparisons among the set of genomes were conducted using GBDP and accurate intergenomic distances inferred under the algorithm 'trimming' and distance formula d_5 [7]. 100 distance replicates were calculated each. Digital DDH values and confidence intervals were calculated using the recommended settings of the GGDC 4.0 [2,7].

Phylogenetic inference

The resulting intergenomic distances were used to infer a balanced minimum evolution tree with branch support via FASTME 2.1.6.1 including SPR postprocessing [8]. Branch support was inferred from 100 pseudo-bootstrap replicates each. The trees were rooted at the midpoint [9] and visualized with PhyD3 [10].

Type-based species and subspecies clustering

The type-based species clustering using a 70% dDDH radius around each of the 11 type strains was done as previously described [1]. The resulting groups are shown in Table 1 and 4. Subspecies clustering was done using a 79% dDDH threshold as previously introduced [11].

Results

Type-based species and subspecies clustering

The resulting species and subspecies clusters are listed in Table 4, whereas the taxonomic identification of the query strains is found in Table 1. Briefly, the clustering yielded 9 species clusters and the provided query strains were assigned to 1 of these. Moreover, user strains were located in 1 of 9 subspecies clusters.

Figure caption SSU tree

Figure 1. Tree inferred with FastME 2.1.6.1 [8] from GBDP distances calculated from 16S rDNA gene sequences. The branch lengths are scaled in terms of GBDP distance formula d_5. The numbers above branches are GBDP pseudo-bootstrap support values > 60 % from 100 replications, with an average branch support of 86.2 %. The tree was rooted at the midpoint [9].

Figure caption genome tree

Figure 2. Tree inferred with FastME 2.1.6.1 [8] from GBDP distances calculated from genome sequences. The branch lengths are scaled in terms of GBDP distance formula d_5. The numbers above branches are GBDP pseudo-bootstrap support values > 60 % from 100 replications, with an average branch support of 97.1 %. The tree was rooted at the midpoint [9].

References

[1] Meier-Kolthoff JP, Göker M. TYGS is an automated high-throughput platform for state-of-the-art genome-based taxonomy. Nat. Commun. 2019;10: 2182. DOI: 10.1038/s41467-019-10210-3

[2] Meier-Kolthoff JP, Sardà Carbasse J, Peinado-Olarte RL, Göker M. TYGS and LPSN: a database tandem for fast and reliable genome-based classification and nomenclature of prokaryotes. Nucleic Acid Res. 2022;50: D801–D807. DOI: 10.1093/nar/gkab902

[3] Freese HM, Meier-Kolthoff JP, Sardà Carbasse J, Afolayan AO, Göker M. TYGS and LPSN in 2025: a Global Core Biodata Resource for genome-based classification and nomenclature of prokaryotes within DSMZ Digital Diversity. Nucleic Acid Res. 2025, gkaf1110. DOI: 10.1093/nar/gkaf1110

[4] Ondov BD, Treangen TJ, Melsted P, et al. Mash: Fast genome and metagenome distance estimation using MinHash. Genome Biol 2016;17: 1–14. DOI: 10.1186/s13059-016-0997-x

[5] Lagesen K, Hallin P. RNAmmer: consistent and rapid annotation of ribosomal RNA genes. Nucleic Acids Res. Oxford Univ Press; 2007;35: 3100–3108. DOI: 10.1093/nar/gkm160

[6] Camacho C, Coulouris G, Avagyan V, Ma N, Papadopoulos J, Bealer K, et al. BLAST+: architecture and applications. BMC Bioinformatics. 2009;10: 421. DOI: 10.1186/1471-2105-10-421

[7] Meier-Kolthoff JP, Auch AF, Klenk H-P, Göker M. Genome sequence-based species delimitation with confidence intervals and improved distance functions. BMC Bioinformatics. 2013;14: 60. DOI: 10.1186/1471-2105-14-60

[8] Lefort V, Desper R, Gascuel O. FastME 2.0: A comprehensive, accurate, and fast distance-based phylogeny inference program. Mol Biol Evol. 2015;32: 2798–2800. DOI: 10.1093/molbev/msv150

[9] Farris JS. Estimating phylogenetic trees from distance matrices. Am Nat. 1972;106: 645–667.

[10] Kreft L, Botzki A, Coppens F, Vandepoele K, Van Bel M. PhyD3: A phylogenetic tree viewer with extended phyloXML support for functional genomics data visualization. Bioinformatics. 2017;33: 2946–2947. DOI: 10.1093/bioinformatics/btx324

[11] Meier-Kolthoff JP, Hahnke RL, Petersen J, Scheuner C, Michael V, Fiebig A, et al. Complete genome sequence of DSM 30083^T, the type strain (U5/41^T) of *Escherichia coli*, and a proposal for delineating subspecies in microbial taxonomy. Stand Genomic Sci. 2014;9: 2. DOI: 10.1186/1944-3277-9-2
